# Evidence for an iterative module in chain elongation on the azalomycin polyketide synthase

**DOI:** 10.3762/bjoc.12.206

**Published:** 2016-10-11

**Authors:** Hui Hong, Yuhui Sun, Yongjun Zhou, Emily Stephens, Markiyan Samborskyy, Peter F Leadlay

**Affiliations:** 1 Department of Biochemistry, University of Cambridge, 80 Tennis Court Road, Cambridge, CB2 1GA, United Kingdom; 2 Key Laboratory of Combinatorial Biosynthesis and Drug Discovery, Wuhan University, Ministry of Education, and Wuhan University School of Pharmaceutical Sciences, Wuhan 430071, People’s Republic of China

**Keywords:** colinearity, ebelactone, enzyme catalysis, marginolactone, natural products, polyketide synthase

## Abstract

The assembly-line synthases that produce bacterial polyketide natural products follow a modular paradigm in which each round of chain extension is catalysed by a different set or module of enzymes. Examples of deviation from this paradigm, in which a module catalyses either multiple extensions or none are of interest from both a mechanistic and an evolutionary viewpoint. We present evidence that in the biosynthesis of the 36-membered macrocyclic aminopolyol lactones (marginolactones) azalomycin and kanchanamycin, isolated respectively from *Streptomyces malaysiensis* DSM4137 and *Streptomyces olivaceus* Tü4018, the first extension module catalyses both the first and second cycles of polyketide chain extension. To confirm the integrity of the *azl* gene cluster, it was cloned intact on a bacterial artificial chromosome and transplanted into the heterologous host strain *Streptomyces lividans*, which does not possess the genes for marginolactone production. When furnished with 4-guanidinobutyramide, a specific precursor of the azalomycin starter unit, the recombinant *S. lividans* produced azalomycin, showing that the polyketide synthase genes in the sequenced cluster are sufficient to accomplish formation of the full-length polyketide chain. This provides strong support for module iteration in the azalomycin and kanchanamycin biosynthetic pathways. In contrast, re-sequencing of the gene cluster for biosynthesis of the polyketide β-lactone ebelactone in *Streptomyces aburaviensis* has shown that, contrary to a recently-published proposal, the ebelactone polyketide synthase faithfully follows the colinear modular paradigm.

## Introduction

Bacterial modular Type I polyketide synthases (PKSs) are multienzymes that govern the biosynthesis of diverse complex polyketide natural products, including clinically useful antibiotics, immunosuppressants, and antitumor compounds. They follow a remarkable assembly-line paradigm, in which each cycle of polyketide chain extension is accomplished by a different set or module of vertebrate fatty acid synthase (FAS)-related enzyme domains [1–4]. The direct connection between the number and type of modules and the chemical structure of the eventual product is often referred to as colinearity. Each module contains a ketosynthase (KS) domain, which recruits the growing polyketide acyl chain from the previous module and catalyses its Claisen-like carbon–carbon bond condensation with the incoming (alkyl)malonyl extender unit, tethered to an acyl carrier protein (ACP) domain. The choice of extender unit installed onto the ACP is dictated by an acyltransferase (AT domain). In addition to these conserved domains, a module may contain ketoreductase (KR), dehydratase (DH) and enoyl reductase (ER) domains that determine the degree and outcome of reductive processing of the newly-formed β-ketoacyl thioester. Finally, the extended chain is passed on to the following module. This processive assembly-line operation, in which all intermediates remain covalently attached to the multienzyme, helps to explain the efficiency of the process. It also neatly explains how the diversity of naturally-occurring complex polyketides is generated by a common biosynthetic mechanism, and provides clues to the evolution of these multienzymes through duplication, capture, deletion, and rearrangement of modules or individual domains [5]. It has both prompted efforts to manipulate PKS domains and modules into novel combinations, as a route to obtaining novel non-natural polyketide products [6–7], and facilitated the discovery of new biosynthetic gene clusters using whole-genome sequence analysis [8–9].

A number of assembly-line PKSs do not exactly follow the modular colinear paradigm, and there is great interest in characterising such exceptions, both for the insights these examples can potentially provide into the catalytic mechanism and specificity of chain extension, and to further our understanding of how these molecular machines have evolved [10–12]. It is clear, for example, that a large number of so-called *trans*-AT PKSs, where attachment of extender units to ACP domains is effected by stand-alone AT enzymes rather than by an intramodular AT domain, have an evolutionary history different from that of canonical (*cis*-AT) modular PKSs [13]. In *trans*-AT PKSs, domains are often found in unconventional order, and modules may be split between different PKS multienzyme subunits. In both types of modular PKS, domains may be present but apparently not used, or expected domains may be missing [10,12]. Perhaps the most striking deviations from colinearity are those where the number of modules in the PKS does not correspond to the number of extension units found in the chemical product [10,12]. Strains subjected either to random mutagenesis or to a targetted block in post-PKS steps have been found to accumulate aberrant products of either PKS module omission ("skipping") or the iterative use of a module ("stuttering") as minor congeners of a product mixture [14–16]. Efficient skipping of an interpolated heterologous module has also been observed in an engineered PKS assembly-line [17–18]. Naturally programmed skipping of a PKS module to make an alternative product is rare, the best-characterised example being the production of both the 12-membered macrolide methymycin and the 14-membered macrolide pikromycin from the same PKS [19], the smaller ring arising from use of an alternative start codon leading to a significantly-truncated final module incapable of condensation. However, an increasing number of PKS systems are now known in which the main product apparently requires iterative use of a module to accomplish two or even three successive rounds of chain extension. First noted in the stigmatellin PKS from *Stigmatella aurantiaca* [20], further examples have been uncovered in the PKSs for aureothin [21–22], borrelidin [23–24], lankacidin [25–26], neoaureothin [27], etnangien [28], crocacin [29], ebelactone [30] and thiolactomycin [31–32]. Given the close mechanistic analogy between fatty acid synthases and an iterative PKS module, it is instructive that a normally colinear extension module of the pikromycin PKS, when studied as a stand-alone protein in vitro, catalyses two rounds of polyketide chain extension [19]. A detailed study of the aureothin and neoaureothin PKSs expressed in a heterologous strain has revealed the role played both by substrate tolerance of the KS domain in the iterative module, and by substrate preference in the KS domain of the downstream module, in favouring longer chains [33–34]. However, much remains to be learned about the mechanism and control of iteration, and the degree to which it influences the structures of complex polyketides.

**Figure 1:**
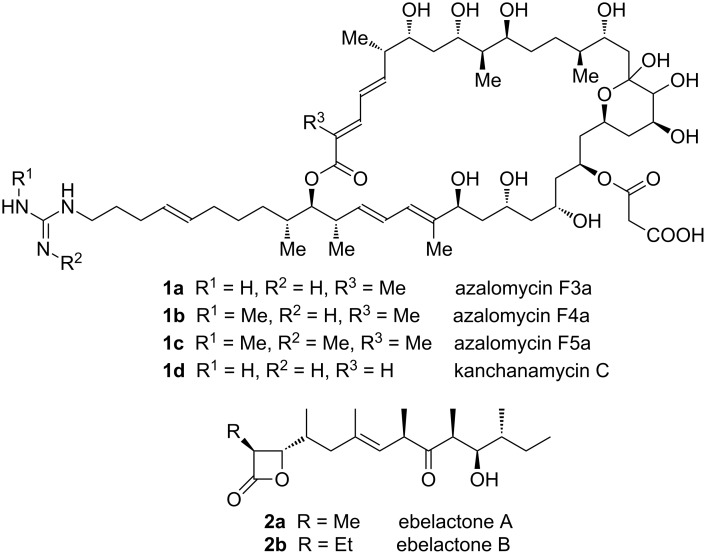
The structures of marginolactones azalomycin and kanchanamycin, and of the β-lactones ebelactone A and B. The stereocentres of azalomycin and kanchanamycin are based on bioinformatic prediction [36].

We aimed to characterise three recently uncovered candidates for iterative operation within a modular PKS, based on sequencing of the structural genes showing fewer modules present in the gene cluster than required to furnish the observed polyketide. Two of these are the near-identical PKSs (Figure S1, Supporting Information File 1) for the antifungal 36-membered marginolactones azalomycin **1a–c** (from *Streptomyces malaysiensis* (formerly *Streptomyces violaceusniger*) DSM4137) and kanchanamycin **1d** [35–36] from *Streptomyces olivaceus* Tü4018; and the third is the PKS for the β-lactone ebelactone, a potent esterase inhibitor from *Streptomyces aburaviensis* **2a,b** (Figure 1) [30]. These examples are particularly interesting as potential model systems because the chemical outcome of the two successive extensions catalysed by the iterative module is predicted to be different (vide infra). We report here that resequencing of the ebelactone modular PKS reveals, surprisingly, that it is fully colinear with no need to invoke iteration. In contrast, the marginolactone PKSs do appear to employ iteration of a single module within the assembly-line.

## Results and Discussion

Ebelactone A (**2a**) has been shown, through incorporation of label from isotopically-labelled precursors, to be constructed from an acetate starter unit and five propionate units [30,37], while ebelactone B (**2b**) arises through the final propionate unit being replaced by a butyrate unit. The ebelactone PKS is therefore expected to contain a loading module and six extension modules. Draft genome sequencing of the ebelactone-producing strain *Streptomyces aburaviensis* ATCC 31860 (NRRL B-2218) was done as described in the experimental section, and the ebelactone (*ebe*) biosynthetic gene cluster (BGC) was located in the sequence using the publicly-deposited sequence [30] as a probe. The organisation of the PKS region was found to differ significantly from that reported previously. The earlier work found seven genes encoding PKS proteins, housing a total of only five extension modules, one of which was split between multienzymes (a very unusual arrangement for a *cis*-AT PKS). To account for the discrepancy, the authors speculated that one module acts iteratively, in the first cycle without reduction of the β-ketoacyl-ACP product, and in the second cycle with reduction of the extended chain to an α,β-unsaturated acyl-ACP. In contrast, we find that the *ebe* PKS cluster contains five PKS genes, the encoded multienzymes together housing one loading module recruiting and decarboxylating malonyl-CoA [38], and six extension modules (Table 1).

**Table 1:** The module arrangement of the ebelactone PKS deduced from genome sequence analysis of *S. aburaviensis*.

Protein	Identity number	Size (aa)	Modules and their domain content	Predicted KR type^a^

EbeA	0356	1055	Loading KS_Q_-AT(a)-ACP	–
EbeB	0357	2204	Module 1 KS-AT(p)-DH-ER^b^-KR-ACP	B1
EbeC	0358	3066	Module 2 KS-AT(p)-KR-ACP- Module 3 KS-AT(p)-KR-ACP	B2 B2
EbeD	0359	3934	Module 4 KS-AT(p)-DH-KR-ACP- Module 5 KS-AT(p)-DH-ER-KR-ACP	B1 B1
EbeE	0360	2178	Module 6 KS-AT(p/b)^c^-KR-ACP-KS^d^	A2

^a^The predicted stereochemical outcome of ketoreduction according to Caffrey [39] and Reid et al. [40], as extended by Keatinge-Clay [41]. KS_Q_, KS-like decarboxylase [38] of the PKS loading module. For explanation of other symbols see the text. ^b^The ER of module 1 has Val at the diagnostic active site position, predicting 2*R* configuration at C12 of ebelactone. The ER of module 5 has Tyr at this position, predicting 2*S* configuration at C4 of ebelactone [42]. ^c^The unusual specificity motif in the extender AT of module 6 (VASH) is consistent with utilisation of either methylmalonyl-CoA and ethylmalonyl-CoA as substrate [43], giving rise respectively to ebelactone A and ebelactone B. ^d^The C-terminal KS may promote formation of the β-lactone and chain-release [30,44].

**Scheme 1:**
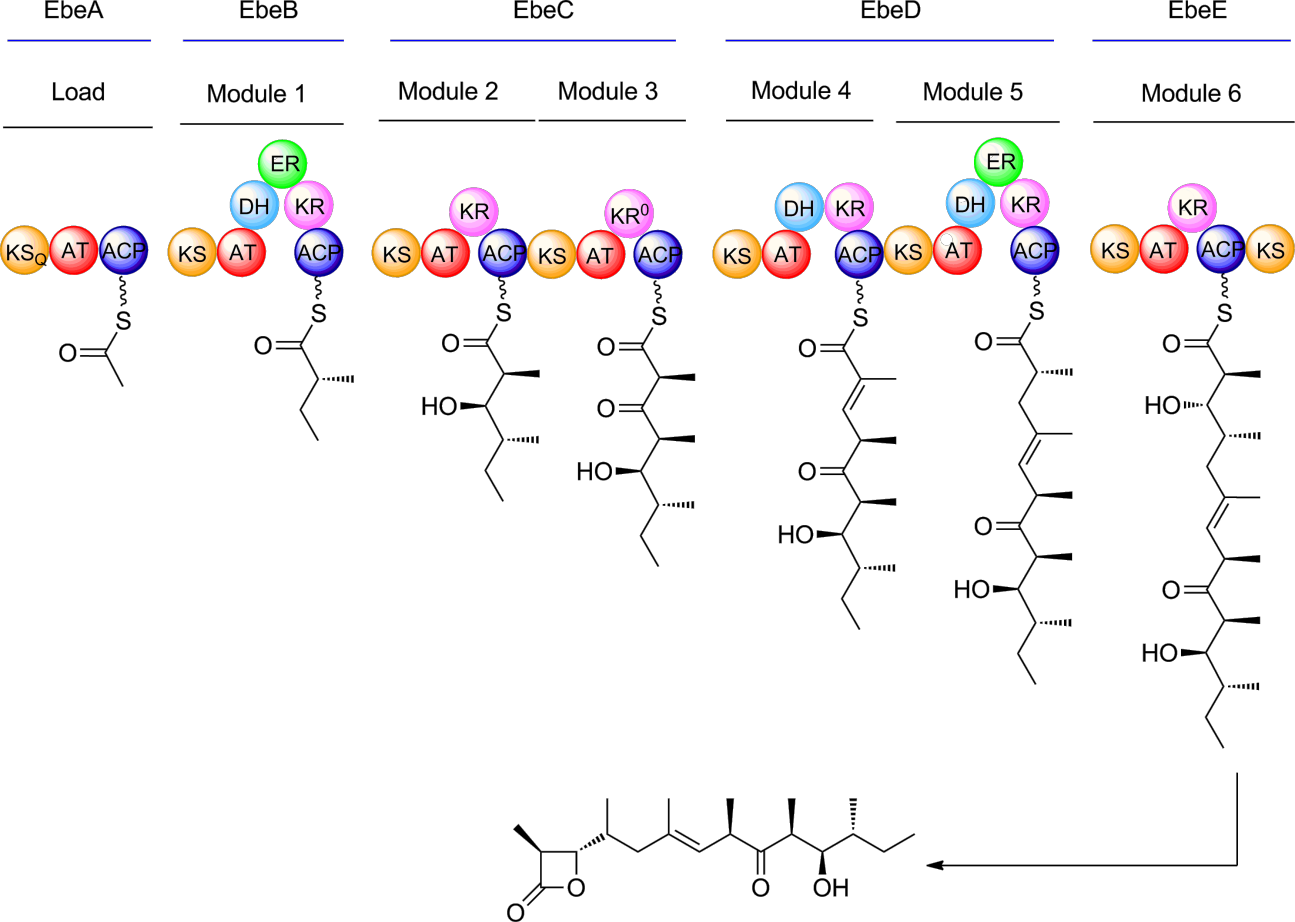
Comparison of the bioinformatic prediction for ebelactone biosynthesis with the known structure of ebelactone A. The only discrepancy is that the ketoreductase domain in module 3 is predicted to be active but is not needed to produce ebelactone.

In fact the domain arrangement within each respective extension module is precisely that needed to generate the full-length linear precursor of ebelactone with the exception that the ketoreductase of module 3 is predicted to be active but is not required for the production of authentic ebelactone. Such skipping of a single, apparently active domain in a *cis*-AT PKS is well-precedented [45–47]. The bioinformatic prediction of the configuration at each of the seven asymmetric centres was also in accord with the configuration of authentic ebelactone confirmed by total synthesis [48] (Scheme 1). The essentially complete congruence between prediction and the known ebelactone structure means, unless the clone sequenced here under the reference number ATCC 31860 is different from that previously analysed under this same ATCC number [37], that the previous proposal of iteration may have been based on mis-assembly of the DNA sequence due to the very high intermodular sequence identity. The ebelactone PKS from our data is faithfully colinear.

**Scheme 2:**
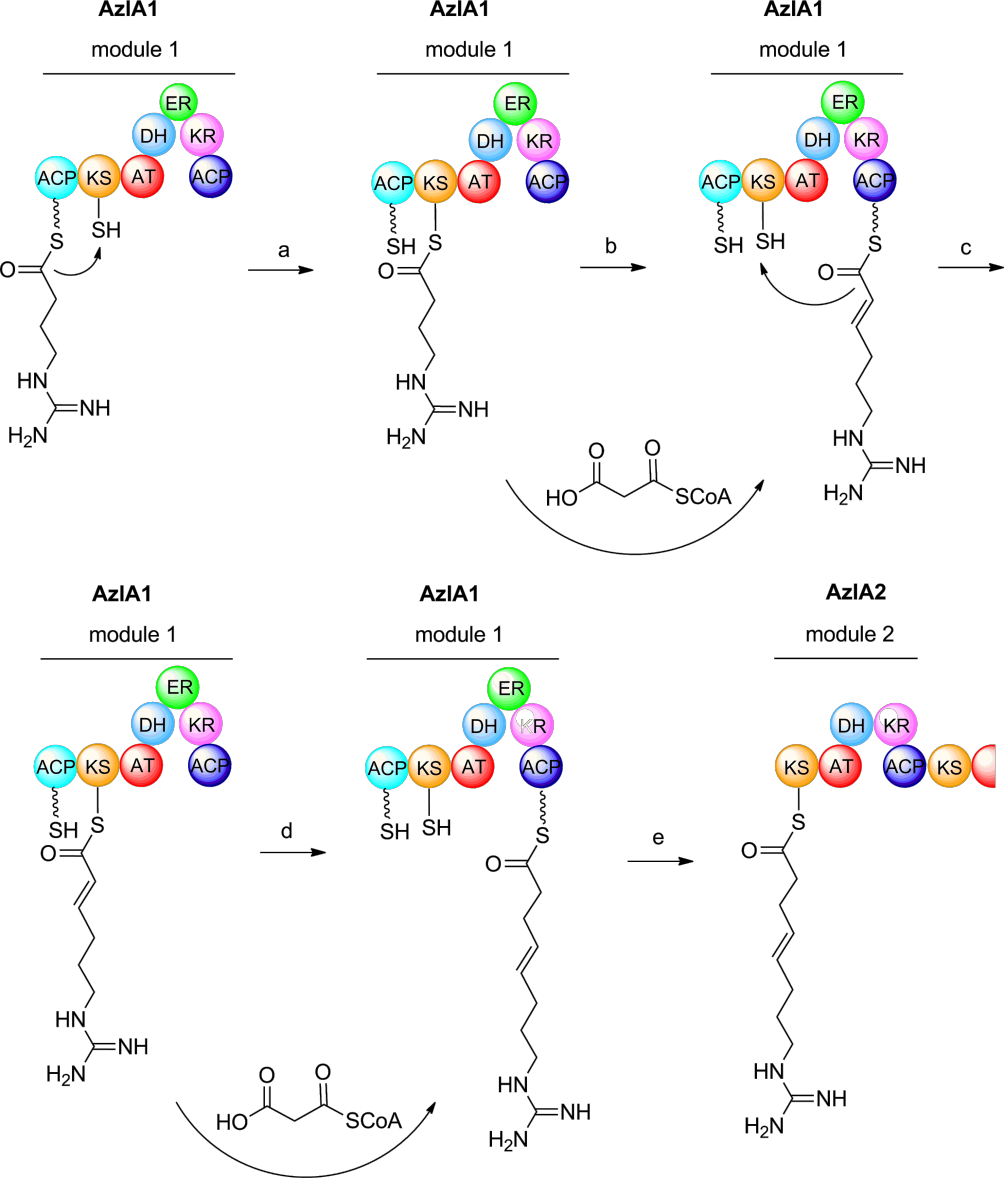
Proposed model for iteration of module 1 of AzlA1 in azalomycin biosynthesis. The 4-guanidinobutyryl starter is loaded onto the loading acyl carrier protein (ACP) by the action of a dedicated acyltransferase (AT) [35]. The starter unit is then transferred from the loading ACP to the ketosynthase (KS) (step a). In the first cycle of chain extension (step b), the starter is condensed with malonyl-ACP and the resulting β-ketoacyl-ACP is then reduced by the action of KR and DH, but the ER domain is "skipped". In step c, the extended chain is back-transferred from ACP to the KS. In the second chain extension (step d), condensation with malonyl-ACP is followed by full reduction. In step e, the triketide chain is transferred to the KS of module 2 of AzlA2.

We have previously reported the DNA sequence of both the azalomycin (*azl*) and kanchanamycin (*kch*) clusters [36]. Unlike ebelactone, these marginolactones undergo late-stage modification, including optional mono- or dimethylation of azalomycin at the guanidino group, and the attachment of a malonyl sidechain at either C-23 or C-25 (Figure 1). No methyltransferase or discrete malonyltransferase is encoded within the respective BGCs. Comparison of the bioinformatic prediction for either marginolactone with the published structure for these molecules [36] reveals that although 20 extension modules are required by the colinear paradigm, only 19 are present. Detailed comparisons showed the discrepancy to be located at the start of chain synthesis, where it appears that extension module 1, housed in PKS multienzyme AzlA1, may catalyse both the first and second cycle of chain extension (Scheme 2). The same is true for extension module 1 of the kanchanamycin PKS, housed in multienzyme KchA1.

In the first cycle, the 4-guanidinobutanoyl starter unit is proposed to condense with malonyl–ACP and the resulting β-ketoacyl–ACP is then reduced by the action of KR and DH but the ER domain is "skipped" so an enoyl thioester is produced. In the second cycle, condensation with a second malonyl–ACP is followed by full reduction, and then the triketide chain is transferred to the KS of the following module, module 2. Module 2 is predicted to catalyse addition of a methylmalonate extension unit and reduction to an enoyl thioester (the module lacks an ER domain). Puzzlingly, the structure of azalomycin requires full reduction at this stage. There is precedent for this, for example in the PKS for the anticancer compound epothilone, where the structure of the product requires reduction to an enoyl thioester by module 4 but this module contains no DH domain [49]. A possible explanation in both cases is that the missing activity is supplied by an enzyme domain in a neighbouring module, but this remains to be demonstrated [10,49].

**Figure 2:**
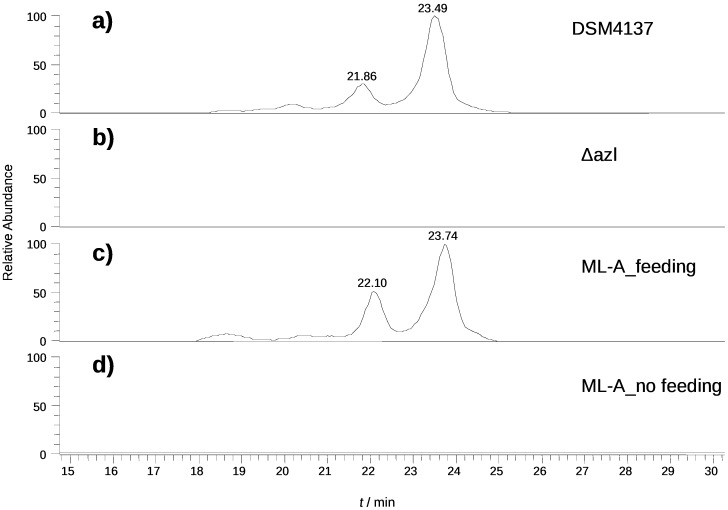
LC–MS analysis of azalomycin F4a production. a) DSM4137 wild type; b) Δazl (*azl* disrupted mutant); c) *S. lividans* ML-A (harbouring the *azl* biosynthetic cluster) fed with 4-guanidinobutyramide; and d) *S. lividans* ML-A not fed with 4-guanidinobutyramide. The two peaks with the same *m*/*z* ([M + H]^+^ = 1082.6) represent azalomycin F4a isomers, differing in the site of attachment of the malonyl group, either at C23-OH or at C25-OH [51].

When the *Streptomyces malaysiensis* (formerly *Streptomyces violaceusniger*) DSM4137 strain was first described, it was reported to produce a different marginolactone called niphimycin (mol wt 1141.7), which compared to azalomycin has an additional propionate unit in the sidechain, as well as other minor differences in the macrocyclic ring [50]. However in our hands azalomycins F3a (**1a**, minor) and F4a (**1b**, major) were produced. The HRMS of the marginolactone from DSM4137 was in agreement with that expected for **1b** (calcd [M + H]^+^: 1082.6734, found 1082.6722). Targetted gene-disruption of the *azl* PKS genes from the cluster (Figures S2 and S3, Supporting Information File 1) led to the specific loss of azalomycin production, confirming the identity of the cluster (Figure 2a and b).

An alternative explanation to the iterative use of a module would be the contribution of additional *azl* PKS multienzymes, encoded by genes located outside the boundaries of the sequenced cluster. In the case of stigmatellin this was ruled out by inactivating all other PKS genes in the genome [52–53] but it can be done more economically by expression of the gene cluster in a heterologous host strain [34,54]. Heterologous production of azalomycin was carried out by expression of the *azl* biosynthetic gene cluster in *S. lividans* TK24. A clone containing the *azl* cluster (~99 kbp), centrally located within a 146.5 kbp insert in the P1-derived bacterial artificial chromosome (BAC) vector pESAC13 [55–56] was isolated from a BAC library of *S. malaysiensis* DSM4137 genomic DNA as described in Supporting Information File 1, and named pYJ2. To allow the introduction of the cloned *azl* cluster into actinomycete host strains that are intrinsically resistant to thiostrepton (*tsr*), the *tsr* resistance cassette of pYJ2 was replaced by the apramycin resistance cassette *aac*(3)IV [57] using RedET recombineering of pYJ2 in *E. coli* to generate BAC clone pML1. Tri-parental mating was used [58] to introduce pML1 into *E. coli* ET12567, a triply methylation-deficient strain that provides higher efficiency of conjugation into many actinomycete strains. To check for significant deletions in clones housing the *azl* genes, PCR primer pairs were designed that would anneal approximately every 10 kbp within the gene cluster. The results were fully consistent with the whole gene cluster being present in *E. coli* ET12567 containing pML1. This strain was used for conjugation with *S. lividans*. Selection for apramycin resistance led to the isolation of exconjugants, four of which were analysed using PCR with two flanking and twelve internal PCR primer pairs. Two colonies were found which appeared to contain the full *azl* gene cluster while the others had apparently suffered deletions at the right end of the cluster. One of the clones harbouring an apparently intact cluster was named *S. lividans* ML-A, and used in further experiments. We have previously shown that the unusual starter unit in azalomycin biosynthesis, 4-guanidinobutanoyl-CoA, requires a dedicated three-enzyme precursor pathway: L-arginine is converted by arginine monooxygenase to 4-guanidinobutyramide, 4-guanidinobutyramide hydrolase converts this to 4-guanidinobutyric acid, and the acid is then activated to 4-guanidinobutyl-CoA by 4-guanidinobutanoate:CoA ligase [35]. The transplanted *azl* cluster only contains genes for 4-guanidinobutyramide hydrolase gene and 4-guanidinobutanoate:CoA ligase [36], and the *S. lividans* genome contains no arginine monooxygenase, so we anticipated that heterologous azalomycin production would require added 4-guanidinobutyramide. Accordingly, *S. lividans* ML-A was grown on SFM agar containing added 4-guanidinobutyramide. After eight days, LC–MS/MS analysis of a methanol extract of the agar plate showed that azalomycin F3a, F4a and F5a were produced. No azalomycins were produced on control plates where 4-guanidinobutyramide was not present (Figure 2c and d). Also, it appears that the heterologous host can supply appropriate, as yet unidentified, enzymes to catalyse methylation of the guanidino group and to attach the malonyl sidechain to the hydroxy group at C-23/C-25. Presumably, these are enzymes acting in primary metabolism and therefore widely present in actinomycete bacteria. Meanwhile, it is clear that the PKS genes in the *azl* cluster as sequenced are sufficient to accomplish the synthesis of the full-length azalomycin chain, without requiring any contribution from additional PKS enzymes encoded elsewhere in the *S. violaceusniger* genome. This finding constitutes strong support for the idea that module 1 of the azalomycin PKS (and by extension its closely-related counterpart in the kanchanamycin PKS) is acting iteratively. In the first cycle, reduction is halted at the enoyl thioester, while in the second cycle full reduction is seen. The identity of the ER carrying out enoylreduction in the third cycle of chain extension remains to be determined. Experiments to determine the detailed mechanism of iteration on the azalomycin PKS are now in progress.

## Conclusion

New exceptions to the so-called colinear rule are of great interest for our understanding of the paradigm of enzyme catalysis used by bacterial modular PKS multienzymes. As pointed out by Moss and colleagues in their review of PKS non-colinearity [10], the observation of iteration emphasises the close mechanistic link between chain extension on (wholly iterative) animal fatty acid synthases and that on bacterial modular polyketide synthases, and it also hints at what could be a major mechanism for the evolution of these processive systems. Nevertheless, the highly repetitive nature of the genes encoding modular PKS makes it easy to misassemble sequence data, and prematurely to diagnose a case of programmed iteration. Thus, the ebelactone PKS, previously reported to be non-colinear [30], in our hands appears faithfully to follow the modular colinear paradigm. In contrast, using heterologous expression of the gene cluster, we have obtained clear evidence for programmed iteration on the first extension module of the PKS for the marginolactone azalomycin. Given the identical gene arrangement in the kanchanamycin gene cluster, we propose that iteration also operates there.

## Supporting Information

File 1 Details of all molecular biological materials and procedures, growth conditions and analytical data.
