# Supplementary material for: Evidence for an iterative module in chain elongation on the azalomycin polyketide synthase
Source: Beilstein J Org Chem. 2016 Oct 11;12:2164–72. doi: 10.3762/bjoc.12.206 (PMC5082578; doi:10.3762/bjoc.12.206)
Supplement: File 1 — Details of all molecular biological materials and procedures, growth conditions and analytical data. [file Beilstein_J_Org_Chem-12-2164-s001.pdf]

Supporting Information

for

Evidence for an iterative module in chain elongation on the azalomycin polyketide synthase

Hui Hong[1], Yuhui Sun[2], Yongjun Zhou[1], Emily Stephens[1], Markiyan Samborskyy[1] and Peter F. Leadlay*[1]

Address: [1]Department of Biochemistry, University of Cambridge, 80 Tennis Court Road, Cambridge, CB2 1GA, United Kingdom and [2]Key Laboratory of Combinatorial Biosynthesis and Drug Discovery, Wuhan University, Ministry of Education, and Wuhan University School of Pharmaceutical Sciences, Wuhan 430071, People's Republic of China

Email: Peter Leadlay* - pfl10@cam.ac.uk

*Corresponding author

Details of all molecular biological materials and procedures, growth conditions and analytical data

1. Supplementary methods

1.1. General analytical procedures

HPLC-MS analysis of *Streptomyces* sp. DSM 4137 secondary metabolites was performed using an HPLC (Agilent Technologies 1200 series) coupled to a Thermo Fisher LTQ mass spectrometer fitted with an electrospray ionization (ESI) source. The HPLC was fitted with a Prodigy 5μ C18 column (4.6 × 250 mm, Phenomenex). Samples were eluted using 20 mM ammonium acetate and MeOH with a gradient of 60% to 95%

MeOH over 30 min at a flow rate of 0.7 mL min^{-1}. The mass spectrometer was run in positive ionization mode, scanning from *m*/*z* 200 to 2000. Production of the polyketides azalomycin F3a, F4a was verified by MS2 on $[M + H]^+$ ions at *m*/*z* 1068.6 and 1082.6 respectively with a normalized collision energy of 30%.

ESI high resolution MS (ESI-HRMS) was carried out on a Thermo Scientific Orbitrap with 60,000 resolution.

1.2. Bacterial strains and culture conditions

Streptomyces sp. DSM4137 was maintained on solid SFM medium (2% mannitol, 2% soya flour, 2% agar). For azalomycin production, strains of DSM4137 and mutant DSMΔazl were cultured in TSBY at 30 °C and 200 rpm in a rotary incubator and harvested after 2–3 days. To check azalomycin production in *Streptomyces lividans* TK24, 6 day TSBY seed culture was inoculated onto SFM agar supplemented with 4-guanidinobutyramide at a concentration of 0.3 mg mL^{-1}. 4-Guanidinobutyramide was prepared enzymatically [1]. The strain was grown at 30 °C for 8 days before analysis. *E. coli* strains were grown in Luria–Bertani (LB) broth (10% tryptone, 5% yeast extract, 10% NaCl) or agar (10% tryptone, 5% yeast extract, 10% NaCl, 2% agar) at 37 °C with appropriate antibiotic selection (kanamycin, at 50 μg mL^{-1}).

1.3. Materials, DNA isolation and manipulation

Bacterial strains, plasmids and oligonucleotides (Invitrogen) used in this work are summarized in Tables S1, S2 and S3, respectively. Restriction endonucleases were purchased from New England Biolabs. T4 DNA ligase and alkaline phosphatase were purchased from Fermentas. All chemicals were from Sigma-Aldrich. Liquid cultures of DSM 4137 for isolation of genomic DNA were grown in tryptone soya broth (Difco). DNA isolation and manipulation in *Streptomyces*, and *E. coli* were carried out using standard protocols [2,3]. PCR amplifications were carried out using a Mastercycler (Eppendorf) and *Phusion* polymerase from New England Biolabs.

1.4. Culture extraction for HPLC-MS analysis of metabolites

1 mL samples of culture broth of either *S. malaysiensis* DSM4137 or mutant DSMΔazl were centrifuged at 20,000*g* for 15 min. The mycelia pellets were then extracted with 1 mL of methanol at 60 °C for 2 hours. The mixture was spun down and the clear methanol extract was evaporated to dryness and redissolved in 200 µL of methanol. 10 µL of the extract was analyzed by LC–MS.

SFM agar plates were extracted with 4.0 mL of methanol at 60 °C for 2 h. Debris was removed by centrifugation, and the organic phase was evaporated to dryness, dissolved in 200 uL methanol, centrifuged, 50 µL of the extract was analyzed by LC–MS.

1.5. Gene disruption in *S. malaysiensis* DSM 4137

To construct the plasmid used for disruption of the *azl* gene cluster, two DNA fragments, a 7417 bp *Bcl*I-*Bam*HI fragment and a 9763 bp *Bam*HI fragment were cloned from cosmids DC10 and 4F8 covering both ends of the azalomycin biosynthetic gene cluster. The two fragments were then inserted into the unique *Bam*HI restriction site of the delivery vector pYH7 [4] with its natural orientation to yield the plasmid pYH10 (Figure S2). The plasmid pYH10 was introduced into DSM4137 by conjugation using ET12657/pUZ8002 as donor host strain on SFM plate. After incubation at 30 °C for 18 h, exconjugants were overlaid with 1 mL water containing 100 µg apramycin and 5 mg nalidixic acid. Single colonies from this plate were transferred to an SFM plate with higher apramycin concentration (12.5 µg mL^{-1}) for further confirmation of antibiotic resistance. Confirmed colonies (Apr^R) were propagated on SFM plate without antibiotic selection to allow the double cross-over to happen. To screen the double cross-over mutants, single colonies from non-selective plate were replicated on an SFM plate containing 12.5 µg mL^{-1} apramycin. The genomic DNA of the candidates with correct phenotype (Apr^S) was isolated and used as PCR template and southern blot. To screen mutant with correct phenotype (Apr^S), a pair of primers azl-1 and azl-2 was used in the 30 µL PCR reaction using a program: initial denaturing at 94 °C for 5 min, followed by 30 cycles at 94 °C for 30 seconds, 55 °C for 40 seconds and 72 °C for 50 seconds, and completed by an additional 5 min at 72 °C. A specific product with the expected size of 473 bp was readily amplified in two individual colonies, while no products were

amplified by PCR when DSM4137 genomic DNA was used as template under the same conditions (Figure S3A). The PCR product was subsequently recovered from gel for sequencing and the result confirmed the 88346 bp DNA deletion between two homologous recombination arms occurred (Figure S2). Meanwhile, southern blot was also performed with the above 473 bp PCR product as a probe. Distinct bands were detected in all genomic DNA samples digested with *Pvu*II (Figure S3B).

1.6. Heterologous expression of azalomycin in *S. lividans* TK24

Heterologous production of azalomycin was carried out by expression of the entire *azl* biosynthetic gene cluster in *S. lividans* TK24. To capture the entire biosynthetic gene cluster, a PAC library (average insert size 145 kb) had previously been generated in *E. coli* DH10B using *Streptomyces malaysiensis* DSM4137 genomic DNA. Large genomic DNA fragments were ligated into pESAC13 vector between two *BamHI* sites. The genomic PAC library was constructed by Bio S&T Inc. (Montreal, Canada) and had been distributed in six 384-well plates (Bio S&T, Montreal). DNA had been extracted from each well then combined in pools of eight colonies to give 3 × 96-well plates of glycerol storage PAC DNA (2304 clones in total).

For screening of the library individual PAC clones were grown in 96 deep-well plates at 37 °C, 300 rpm overnight. For each row (8 wells), the cultures (800 μL) were pooled into a 15 mL plastic centrifuge tube (Greiner) and centrifuged (4,600*g*, 10 min, 4 °C). After DNA purification by alkaline lysis PCR reactions with primer pairs (aza-1F, aza-1R) complementary to the centre region of the azalomycin biosynthetic cluster were performed. Those samples that gave a band of the correct size were subjected to another round of PCR – with 2 pairs of primer, complementary to the left end (aza-Lf1, aza-Lr1) regions of the cluster and right end (aza-Rf1, aza-Rr1) regions of the cluster. Clones corresponding to the positive hits were grown, DNA was isolated from each individual clone and subjected to end-sequencing with the universal primers of SP6 and T7. The positive clone, named pYJ2, showed the *azl* gene cluster was centrally located within a 146 kb insert with 21 kb and 26 kb of flanking sequence to the right and left of the gene cluster respectively. To allow introduction of the cloned *azl* cluster into actinomycete strains that are intrinsically resistant to thiostrepton, the *tsr* resistance cassette of pYJ2

was replaced by the apramycin resistance cassette *aac(3)IV*, by using GeneBridges' Quick and Easy BAC Modification protocol based on Red/ET recombination. PCR and sequencing confirmed that the *aac(3)IV* had inserted at the correct site, replacing the *tsr* cassette. This newly-generated PAC clone was named pML1.

1.7. *E. coli* triparental mating and conjugation of *S. lividans* TK24

E. coli cells DH10B/pML1 (Apr^R), TOPO10/pR9604 ($Carb^R$) and ET12567 (Cam^R) were inoculated into 5 mL LB medium containing appropriate antibiotic and incubated overnight at 37 °C, 250 rpm. From the overnight culture 500 μL was inoculated into 10 mL LB medium containing half of the working concentration of appropriate antibiotic and incubated at 37 °C, 250 rpm until A_{600} reached 0.4. The cells were harvested by centrifugation at 2,200*g* for 5 min and washed twice with 20 mL of LB medium. The supernatant was discarded and the pellet was resuspended in 500 μL of LB medium. 20 µL of each strain was dripped onto the same location on the LB agar plate lacking antibiotics so that the three strains were mixed together. After drying, the plates were incubated at 37 °C overnight for tri-parental conjugation. Next day, to select for *E. coli* ET12567 derivatives containing the PAC clone and the helper plasmid (pR9604) the cells from the spot were streaked onto fresh LB agar plates containing apramycin, chloramphenicol and carbenicillin antibiotics and incubated at 37 °C overnight. Single colonies were used to inoculate LB medium containing the antibiotics. To test the integrity of *azl* cluster in the ET cell, twelve PCR primer pairs (AZA1-12) with annealing sites approximately every 10 kb and unique within the *azl* cluster were designed. PCR analysis was carried out using the two flanking and twelve internal primer pairs to confirm the presence of the intact PAC clone in ET12567 cells.

Conjugation between *E. coli* ET12567 containing pML1 and the helper plasmid (pR9604), and *S. lividans* TK24 strain was carried out according to the protocol as described by Kieser et al. [3]. Mixtures of *Streptomyces* and *E. coli* were plated on SFM agar plates, and overlaid after 16 h with apramycin (5 µg mL^{-1}) and nalidixic acid (25 µg mL^{-1}). The putative ex-conjugates were streaked onto an SFM plate containing aparamycin (50 µg mL^{-1}) and nalidixic acid (25 µg mL^{-1}). Apramycin resistant colonies were grown in TSBY liquid medium for genomic DNA purification. PCR analysis was carried out with

two flanking and twelve internal primer pairs. One of the clones harbouring the intact cluster was named *S. lividans* ML-A, and used in further experiments.

1.8. Genome sequencing of *Streptomyces aburaviensis* ATCC 31860

Nextera shotgun and Nextera mate-pair libraries were constructed from high molecular weight genomic DNA isolated from *S. aburaviensis* ATCC 31860 using the manufacturer's protocol. Sequencing was carried out on an Illumina MiSeq platform using the Illumina V2 500 in 2 × 250 bp mode. Reads processed using a custom adapter trimming tool (fastq_miseq_trimmer). Read pairs were then preassembled using FLASH v1.2.11 (https://ccb.jhu.edu/software/FLASH). Several assemblies were carried out using either all or subsets of the input dataset, and the best assembly was selected using a score calculated from scaffold N50, edge and total number of contigs. ORFs were predicted ab initio using a customised version of the FGENESB pipeline V2.0 (2008) [www.softberry.com] and blasted against filtered NCBI NR and KEGG datasets. Customised linguistic analysis was used for transfer of gene annotations. Annotation results were saved in EMBL format and manually curated in Artemis (http://www.sanger.ac.uk/science/tools/artemis). The revised sequence of the ebelactone polyketide synthase genes has been deposited in the European Nucleic Acid Archive under accession number LT608336.

.

2. Supplementary Figures

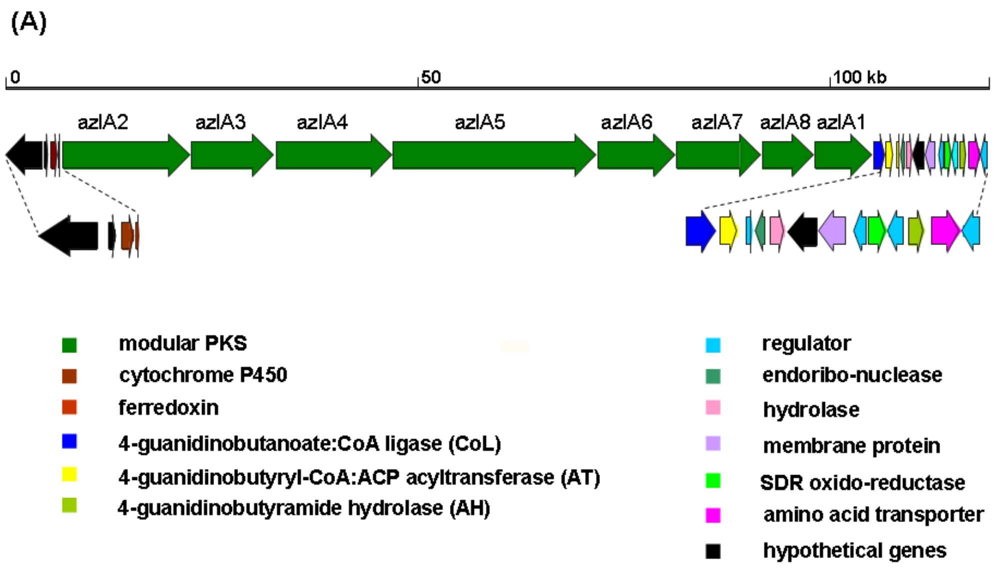

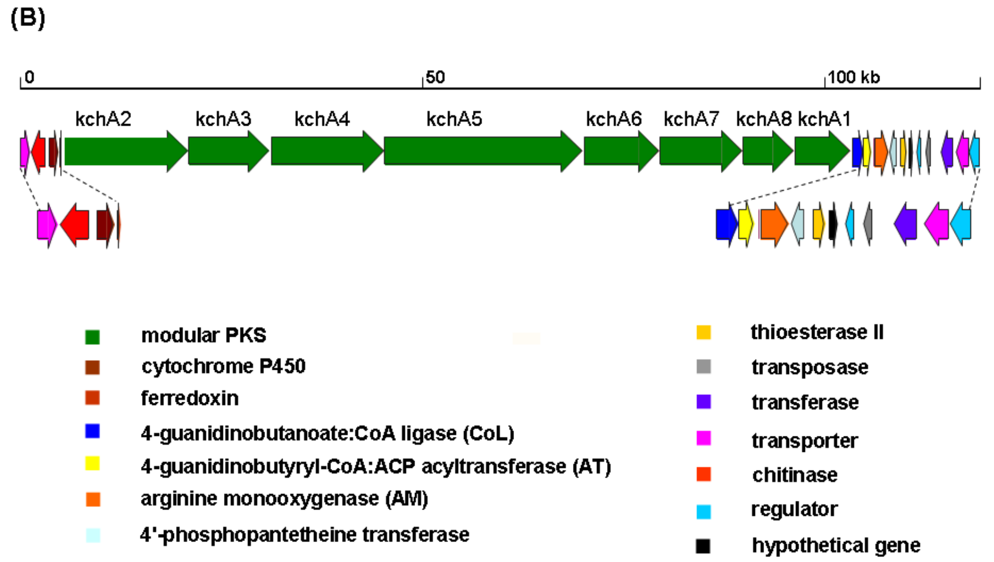

Figure S1: The organization of the biosynthetic gene clusters: A) for azalomycin in *S. malaysiensis* DSM 4137 and B) for kanchanamycin in *S. olivaceus* Tü 4018, and assignment of putative gene functions.

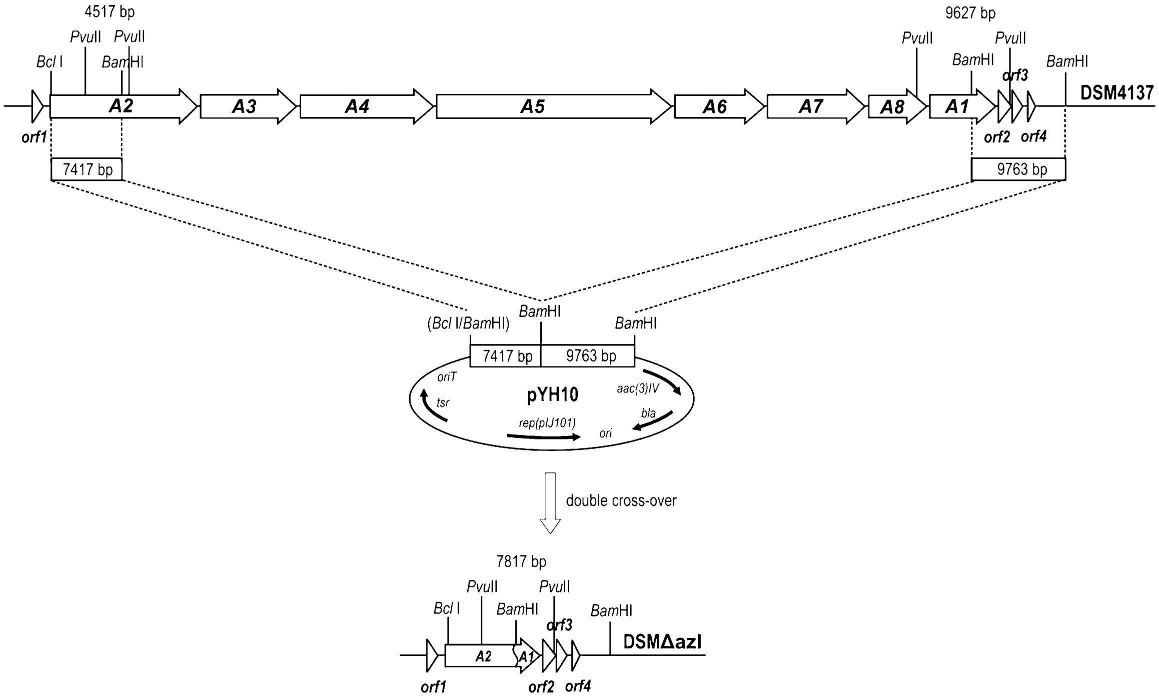

Figure S2: The organization of the azalomycin biosynthetic gene cluster before and after deletion. The numbers between *Pvu*II sites represent the expected size of the fragments hybridizing to the probe (473 bp PCR product using primers azl-1 and azl-2) after digested with *Pvu*II.

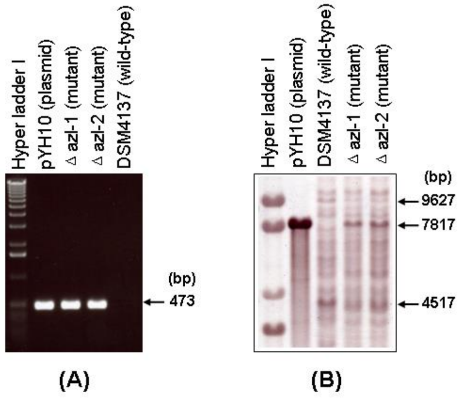

Figure S3: Confirmation of gene disruption by PCR (A) and Southern blot (B). A pair of primers azl-1 and azl-2 flanking deleted region was used for quick screening to identify double cross-over mutants. The plasmid pYH10 used for disruption of the *azl* cluster and chromosomal DNA isolated from wild-type and mutants were digested with *Pvu*II.

3. Supplementary Tables

Table S1: Bacterial strains used in this study.

Strain	Genotype/Characteristics	Reference
E. coli		
DH10B	F^{-} mcrA Δ(*mrr*-*hsd*RMS - *mcr*BC), Φ80*lac*ZΔM15, Δ l*ac*X74 *rec*A1 *end*A1 *ara*D139 Δ (*ara leu*)7697 *gal*U *gal*K *rpsL nup*G λ– host for general cloning	Invitrogen
E. coli DH10B/PAC library	Contains DSM4137 PAC library clones *neo, tsr*	Bio S&T, Montreal
E. coli DH10B/pML1	Contains PAC clone with *azl* gene cluster, *neo, tsr*	This research: PAC modification
E. coli TOPO10/pR9604	Helper strain in triparental mating	[5]

ET12567	(F$^-$ *dam*-13::Tn9 *dcm*-6 *hsd*M *hsd*R recF143 zjj-202::Tn10 *gal*K2 *gal*T22 *ara*14 *pac*Y1 *xyl*-5 *leu*B6 *thi*-1)<br>Donor strain for conjugation between *E. coli* and *Streptomyces* in triparental mating	[6]
S. malaysiensis DSM4137	Azalomycin-producing strain	[1]
DSM4137 Δazl	*azl* pks genes deletion mutant strain	this work
Streptomyces lividans TK24	Heterologous host for azalomycin production	[7]
S. lividans ML-A	Heterologous host with integrated into the genome the entire azalomycin biosynthetic gene cluster	this work

Table S2: Plasmids used in this work.

Plasmid	Genotype/Characteristics	Reference
pYH7	*E.coli-Streptomyces* shuttle vector	[4]
pYH10	*azl* pks genes disruption construct in which a 88346 bp internal fragment comprising of *azl* pks genes was deleted	this work
pESAC13	*bla, neo, tsr, parA, parB, sacB,oriT*<br>PAC library	[5]
pIJ773	*aac(3)IV*	[8]
pYJ2	pESAC13 based PAC vector with cloned azalomycin biosynthetic gene	this work
pML1	the *tsr* resistance cassette of pYJ2 was replaced by the apramycin resistance cassette *aac(3)IV*	this work

Table S3: Oligonucleotide primers used in this work.

Primer	Nucleotide sequence (5' to 3')
Primers used for PAC library screening	
aza-1F	TCTGGCCTCCTTTCATTCTTCAGTC
aza-1R	TCGAGACCGAAGGTGTACGAGATAC
aza-Lf1	AACAAGAGCGGGAACAGGGACAG
aza-Lr1	AGTCTCAACTTCTCGCGGTGGTAC
aza-Rf1	TCTGGACGCCTATATGGAAGGTCTC
aza-Rr1	TCTGCTCACCGATGTCAGTTTCGAG
AZA-1f	TCGACGGGGAGCGCAGTTCCTTCTC
AZA-1r	TTCACCAACCAGCCCTGGCAGCTG
AZA-2f	ACGAACGCGCATGTGATCCTGGAG
AZA-2r	ACCCTCAACACCACGCAACACAC
AZA-3f	TCACGGTGGTTCCCTGGCTGCTCTC
AZA-3r	TGAACGCCGTCTGGTTCAGCAACTC
AZA-4f	TGTTGTTGGAAGTGTGGAGCTACTG

AZA-4r	AACTCGTCGGCGTCGTAGAGGAAG
AZA-5f	TCCAGAAGGTGGCGGATACATGGAC
AZA-5r	AATATCGATTCCCAGCCCACCG
AZA-6f	TCGGTGGAGTGGGAGTCCGTCTTC
AZA-6r	ACGTGGTAGCGCCAGTTGTCGAT
AZA-7f	AGCCTCGCGGCAGCGTTCTGATC
AZA-7r	TCCGGCGTAGGAGGAGAAGAGGATG
AZA-8f	ATCTTCATCGAGGTGAGCCCGCATC
AZA-8r	TGGTCGCGGTTGTTGCGGTGATAC
AZA-9f	AACGCGGGCGTGACCAGGTTCTTC
AZA-9r	AGCACCTCGGTCAGCGAGGCGTTTC
AZA-10f	ATGACTCGCTCTTCCGGCTGGACTG
AZA-10r	AACACCAGGTAGGAGTCGTCGAA
AZA-11f	ATCGTCGGGATGAGCTGCCGTTTC
AZA-11r	TCGAAGCCGAAGGTGTAGGAGATG
AZA-12f	TGGACATGGTGCTGGACTCGCTC
AZA-12r	CGCGCCGCATGGTGAACACGTTCTT
Sp6	TATTTAGGTGACACTATAG (for sequencing only)
T7	TAATACGACTCACTATAGGG (for sequencing only)
Primers for amplification /confirmation of apramycin resistance cassette	
AprR_F	AGGCGAATACTTCATATGCGGGATCGACCGCGCGGGTCCC GGACGGGGAACTGACGCCGTTGGATACACCAAG
AprR_R	ATCACTGACGAATCGAGGTCGAGGAACCGAGCGTCCGAGG AACAGAGGCGCTTATGAGCTCAGCCAATCGACTGG
Confirm_APR	TCAACTGGGCCGAGATCCGTTGA (for sequencing only)
Re-confirm_APR	AGCTGACCGATGAGCTCGGCTTTTC (for sequencing only)
Primers for confirmation of phage 31 attachment site	
attL_F	ACGATGTAGGTCACGGTCTCGAAG
attL_R	ATGACGTTTCCCTGCCGGAAGAC
attR_F	AGGACGGGTGTGGTCGCCATGATC
attR_R	CATCATGATGGACCAGATGGGTGAG
Primers for PCR screening of DSM4137 Δazl mutants	
azl-1	GGGTTCCGTCGTGGTGTC
Azl-2	GAGGTTGTCCAGGGTGCC

References

[1]. H. Hong, T. Fill, P. F. Leadlay, *Angew. Chem., Int. Ed.* **2013**, *52*, 13096 – 13099.

[2]. J. Sambrook, D. W. Russell, Molecular Cloning: A Laboratory Manual, 3rd ed. Cold Spring Harbor Laboratory Press, New York, **2001**.

[3]. T. Kieser, M. Bibb, M. Buttner, K. F. Chater, D. A. Hopwood, Practical *Streptomyces* Genetics. The John Innes Foundation, Norwich, **2001**.

[4]. Sun, Y.H., He, X.Y., Liang, J.D., Zhou, X.F., and Deng, Z.X. (2008). Analysis of functions in plasmid pHZ1358 influencing its genetic and structural stability in *Streptomyces lividans*1326. *Appl. Microbiol. Biotechnol. 82*, 303-310.

[5]. A. C. Jones, B. Gust, A. Kulik, L. Heide, M. J. Buttner, M. J. Bibb, *PLoS ONE* **2013,** *8,* e69319.

[6]. D. J. MacNeil, K. M. Gewain, C. L. Ruby, G. Dezeny, P. H. Gibbons, T. MacNeil, *Gene* **1992,** *111*, 61-68.

[7]. Hopwood D, Kieser T, Wright H & Bibb M (1983) Plasmids, Recombination and Chromosome Mapping in *Streptomyces lividans* 66. *Journal of General Microbiology* **129:** 22257–2269.

[8]. Gust B, Challis GL, Fowler K, Kieser T & Chater KF (2003). PCR-targeted *Streptomyces* gene replacement identifies a protein domain needed for biosynthesis of the sesquiterpene soil odor geosmin. *Proc Natl Acad Sci USA* **100**:1541–1546.
